# New insights in Microbial Fuel Cells: novel solid phase anolyte

**DOI:** 10.1038/srep29091

**Published:** 2016-07-04

**Authors:** Tonia Tommasi, Gian Paolo Salvador, Marzia Quaglio

**Affiliations:** 1 Center for Sustainable Futures, Istituto Italiano di Tecnologia, C.so Trento 21, 10129 Torino, Italy

## Abstract

For the development of long lasting portable microbial fuel cells (MFCs) new strategies are necessary to overcome critical issues such as hydraulic pump system and the biochemical substrate retrieval overtime to sustain bacteria metabolism. The present work proposes the use of a synthetic solid anolyte (SSA), constituted by agar, carbonaceous and nitrogen sources dissolved into diluted seawater. Results of a month-test showed the potential of the new SSA-MFC as a long lasting low energy consuming system.

Renewable energy sources (RES) are important contributors to the transition to a low-carbon society, being able to mitigate emissions of greenhouse gases, lower other environmental pressures associated with conventional energy production and overturn the dominance of fossil fuels^1^. Among the several RES, developed and under development, microbial fuel cells (MFCs) have emerged, during the last decade, as one of the most versatile and promising one. A key reason that could make the MFCs a strategic technology is related to their working principle: they can generate electricity through the catalytic activity of exoelectrogenic bacteria involved in the anaerobic oxidation of organic substrates acting as low grade fuels^2^,^3^,^4^. The amount of energy that can be obtained by MFCs is relatively low if compared to other fuel cell technologies^2^, but they have a unique feature in the field of fuel cells: they can harvest chemical energy from several classes of wastes, with the potential to effectively and directly convert into electrical energy several non-purified organic substrates, naturally present in different environments. Moreover, operating at room temperature and pH close to neutrality^2^,^5^,^6^ MFCs could be one of the most interesting technology for application in remote areas.

Despite for their terrific potential, only relatively few works have tried to demonstrate that MFCs can be used as real power sources, as the pioneering research on MFCs integration on robotic platforms as the solely power source^7^, and the demonstration of MFCs to power electronic systems^8^. Indeed, in the most part of current applications, focus is more given to strategies that can exploit other useful properties of microbial fuel cells, such as waste water treatment or the production of valuable chemicals^9^, combining them to electricity generation in order to increase the overall efficiency of the system. These approaches are advantageous if big plants are designed, while strategies are currently needed to boost research on small scale MFCs, more emphasizing their key potential of being easily movable from one place to another (i.e. portable) and potentially able to operate for long time, neither with the need to be connected to the power grid, nor to the need of human intervention for replenishment (i.e. autonomous)^9^.

In MFC technology, autonomy can be directly related to the density of the organic matter present into the substrate used to feed the cells, and on its availability over time: the higher the latter parameters, the better the performance and the lifetime of the MFC. Firstly introduced in 2001^10^,^11^ using marine sediment as the source of both bacteria and the organic matter, sediment microbial fuel cells (SMFC) demonstrated, for the first time, the possibility to use solid phase organic matter at the anode and they are, up to now, the only class of MFCs able to work autonomously for very long time^12^. SMFC can be designed both as large scale ones, to be used in sea environment, or as small scale, portable systems^13^. Starting from that first example, the concept of SMFC has been extended to the use of other solid phase anolytes as soil in plant MFCs^14^ and urban solid-wastes^12^,^15^, being more generally referred to as solid phase MFC^11^,^15^,^16^ (SPMFC) even if the studies disregarding many often from the concept of portable systems, considering that they often require feeding pumps^17^. In this communication we propose a new approach to the design of small size microbial fuel cells in which the liquid anolyte containing carbonaceous and nitrogen sources is substituted by a new solid one, named synthetic solid anolyte (SSA), obtained by means of the addition of agar. The key idea is to convert the liquid anolyte into a solid one, able to trap the nutrients, granting an increase of their density and a slow release over time as it happens in natural solid phase anolytes. We used the bacteria compatible Agar as the gelation agent, actually converting into a gel the liquid, seawater based, anodic substrate. We placed the solid anolyte into two chambers reactors, and monitored the performance of the resulting MFCs in terms of the open circuit voltage, short circuit current and power output. While traditional liquid electrolytes need to be frequently replaced, the SSA-MFC showed a lifetime of 4 months without the need of human assistance.

## Results

### Solid phase anolyte

**Figure 1:**
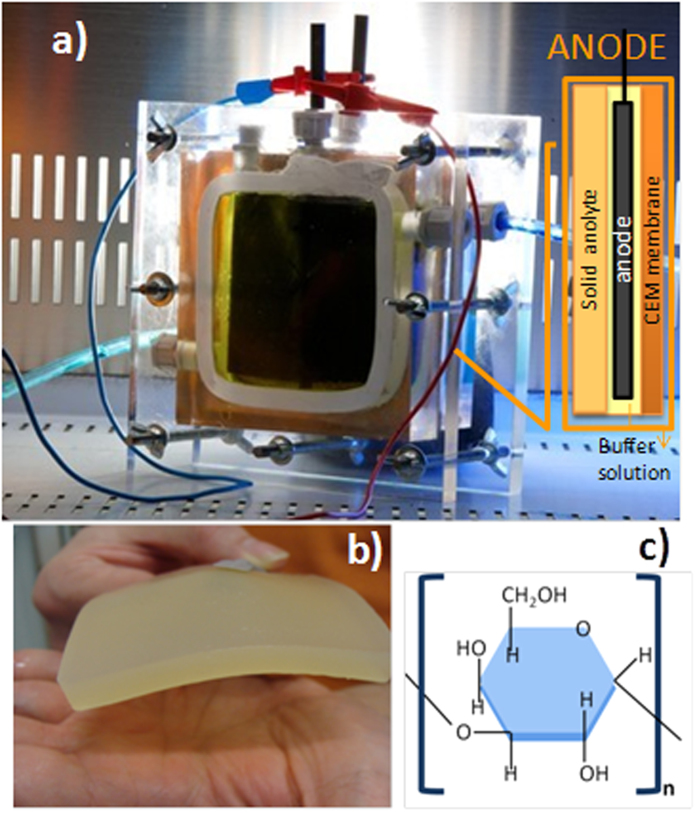
(**a**) The two chambers MFC used in this work: the inset shows the distribution profile of SSA inside the anode chamber: the anodic electrode was lay down on SSA and immersed into buffer solution and sandwiched between SSA and CEM membrane; (**b**) a picture of the agar based solid phase anolyte before use: respect to traditional anolyte it embedded higher energy density and acted as a true energy storing system for bacteria metabolisms and (**c**) the molecular structure of agar, gelling agent that permits to obtain SAA.

A solid phase anolyte has been prepared mixing reagents that are common nutrients for bacteria, such as glucose, fructose, peptone and sodium acetate, with a gelling agent such as agar (Fig. 1c). Agar has been chosen because it is a natural polysaccharide, extracted from a group of red-purple marine algae, well-known in biology as a culture medium of bacteria^18^, glucose and fructose, beyond to be fundamental nutrient for bacteria, can act as cross-linking molecules during agar gelification giving a more compact solid and a less shrinkage in case of dehydration^19^.

The gel has been prepared using a phosphate buffer solution, for preventing an acidification of the anodic chamber that would be detrimental for the bacteria life.

A conductivity measurement of SSA has been performed in order to test the ion mobility inside the gel matrix. The obtained value, 31.0 mS/cm, is not far from the result for the liquid electrolyte (27.6 mS/cm) having the same composition save for agar.

Once the SSA was put into the cell, it was hydrated by means of the phosphate buffer solution. No further liquids, with or without nutrients, has been added during the experiments.

### Device performance

The experiments have been conducted in Microbial Fuel Cells consisting in two squared chambers: the anode and the cathode, separated by a cation exchange membrane, as shown in Fig. 1a.

In the anodic chamber, the solid phase anolyte (Fig. 1b) fills the chamber for its whole volume, save for the space necessary to place the electrode immersed into phosphate buffer solution. The cathodic chamber has been filled by a liquid electrolyte (a phosphate buffer solution of potassium ferricyanide).

Among the experiments that were carried out, the results of the most complete in terms of characterization and longer time span are presented. From the electrical point of view, the experiment has been divided into two phases. The first phase was aimed at understanding the cell behavior under a constant low stressing load condition, while permitting the establishment of a stable electroactive biofilm at the anode^20^. In the second phase, from day 15 to day 30, fifteen loads, from 330 to 8800 Ohm, from the highest to the lowest were sequentially applied, in order to evaluate the performance of the cell covering different classes of applications.

Moreover, during both phases, the applied resistance was removed in order to perform electrochemical characterizations such as Linear Sweep Voltammetry (LSV) and open circuit voltage measurements, after stabilization period of open circuit voltage (OCV).

**Figure 2:**
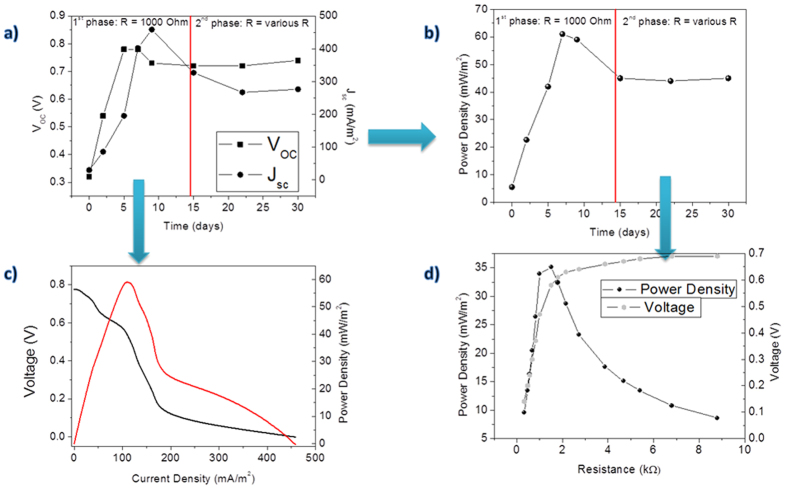
(**a**) Mean value of open circuit voltage (Voc) and current density (Jsc) recorded during the electrochemical characterizations over time; (**b**) maximum power densities derived from the LSV curves; (**c**) example of a LSV curve, collected in the 1^st^ phase (day 7) and (**d**) power densities and voltages measured under the application of external loads during the 2^nd^ phase of the experiment (from day 15 to 30).

Results are shown in Fig. 2. As regards the cell performance in the first phase of the experiment, under the 1000 Ω load, the recorded cell voltage was about 0.5 V, with a contribution of the anodic potential of about −0.3 V vs Ag/AgCl (−0.1 V vs SHE).

This behavior demonstrates that the presence of the solid state electrolyte did not inhibit the establishment of the proper reductive conditions at the biofilm/anodic material interface, with electrons being properly released to the anode. The left side of Fig. 2a, obtained by polarization techniques, clearly shows that after a stabilization period the V_OC_ and J_sc_ values steadily increase, reaching the best values of about 0.8 V and 0.5 A/m^2^ respectively. In the second phase, reported in the right side of Fig. 2a, the values of both V_OC_ and J_sc_ slightly decrease, but are quite stable. The power density trend has the same behavior that was noticed for V_OC_ and J_sc_, as shown by Fig. 2b. The maximum power density value is closed to 60 mW/m^2^ recorded during day 7 (Fig. 2b,c). Interestingly, in the second phase, even if the biological system was exposed to more stress because of the several loads applied in a relatively short time, the output power density was quite stable and close to 45 mW/m^2^ (Fig. 2c). The average power density and potential difference referring to each applied loads from day 15 to 30 are shown in Fig. 2d. At both the highest and the lowest resistances (i.e., 8000 Ω and 330 Ω respectively), the minimum power density values were obtained, that are 9.4 and 9.56 mW/m^2^ respectively. This behavior can be explained considering the proximity of those values to OCV and SCC conditions, with potential difference values of 690 mV and 140 mV respectively. For all values between 820 and 2150 Ω the power densities reach the highest values, with the maximum point of 35 mW/m^2^ at 1500 Ω, with a voltage of 580 mV and a current density of 61 mA/m^2^.

### Energy efficiency of the SSA-MFC

Once the 30 days experiment was over, the MFC was opened and the electrolyte was extracted. A provision of the possible MFC lifetime has been calculated by comparison of the initial energy content in SSA and the energy produced, by MFC, during the experiment. The energy obtained after one-month of operation running, named E_MFC-OUTPUT_, was calculated starting from the electricity generated by applying loads during the time-course of the experiment, as described in Methods.

Instead, an estimation of the energy embedded into SSA before (E_initial_) and after one-month operation (E_final_) was made by calorimetric data, in terms of lower heating value. Therefore, the energy consumption (E_consumed_) by means of microorganism’s metabolism could be deduced by the difference between E_initial_ and E_final_. The energy recovery from 1-month experiment under loads is called E_MFC-OUTPUT._

**Figure 3:**
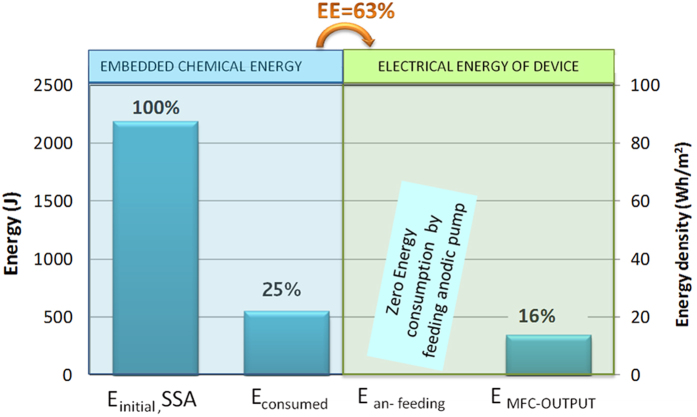
Energy evaluation of SSA-MFC: from the initial energy embedded into SSA (2013 J), to the energy consumed (503 J), till the energy recovery by applying resistances over 30 days period (E_MFC−OUTPUT_). The right axis shows energy density values in Wh/m^2^. The values of energy are also shown in percentage, in comparison to the initial energy present in SSA (100%). Energy efficiency, that is the ratio of the useful energy recovered by MFC device to the heat of combustion of the SSA consumed, is about 63%.

In order to estimate the energy really available for users, the total energy spent to run the device should be taken into account. In this system, only cathodic replacement has an energy expenditure, considering that the SSA-MFC device does not require in this configuration anodic feeding, mixing and temperature heating. The next challenge will be to move toward a complete autonomous system thanks to energy and environmental sustainable cathode, such as the air-cathode that will be coupled with the SSA^21^,^22^. Figure 3 depicts the energies involved in SSA and their ratio respect to the energy embedded in SSA, considered hence as 100%.

As shown in Fig. 3, only a quarter of energy was consumed during the 30 days experiment (E_consumed_ = 503 J) and therefore, it was possible to estimate a total working time of the SSA-MFC of at least 4 months in continuous working condition as a self-sustainable energy harvester.

Taking into account the E_MFC-OUTPUT_ from 1-month experiment under loads (~251 J) and the chemical energy consumed by bacteria in that period (~503 J), it was possible to evaluate the energy efficiency (EE) of the SSA-MFC that reaches 63% (details of the used formula in Methods).

## Discussion

The gel prepared as described in the method paragraph, can be considered a solid state electrolyte, even if inside its framework a liquid phase is still present, because it is well accepted by the scientific community considering gels as solid electrolytes^23^,^24^.

In order to estimate the resistances of ions conductions inside the solid electrolyte, the ohmic resistance has been evaluated. The estimation of the Ohmic losses (R_Ω_) arising, among other factors, from the resistance of ions conduction into the electrolyte and the membrane, and of electrons conduction through the electrodes^25^. It was carried out by means of the current interrupted method (CIM), as described in Methods (MFC operation and characterization). The average value of (12 ± 2) Ω was calculated considering resistances from both the first and the second phase. This result is quite interesting and helps in clarifying that the presence of the new introduced solid electrolyte does not increase the R_Ω_ value. The obtained Ohmic losses are, comparable with those typically measured for liquid anolyte with the same boundaries conditions^26^,^27^. Once the experiment was over it was possible to notice that the SSA was less compactness than it was at the beginning of the experiment. This phenomenon can be explained by the contribution of two different processes: one can be related to the glucose and fructose consumption due to the bacteria, that causes the reduction of the cross-linking degree among the agar molecular chains, while the other processes can be related to an agar degradation due to the seawater bacteria inoculum, because many marine microorganisms can degrade this algal compound by production of agarase enzyme. In particular the presence of craters on the SSA surface, observed at the end of experiment is a result of the enzymatic digestion of the agar^18^.

The EE, defined as the ratio of energy recovered by the MFC to the heat of combustion of the consumed organic substrate^28^, gives important information on the energetic performance of the device. Moreover, since it takes into account energy losses due to external devices, such as the pumps for feeding and recirculation^28^, it can help appreciating the unique features of the SSA-MFC. Indeed, not needing hydraulic pumping systems for anodic feeding, the SSA-MFC has a zero energy contribution from external systems at the anode, helping to obtain an overall positive energy balance^29^, with an impressive value of EE close to 63%. It is important to say that since this study was mainly focused to analyze the behavior of the new SSA, and hence at this stage the repletion of catholyte, in terms of energy costs, has not been taken into account, because the focus is on the anodic chamber energy balance and not that of the whole MFC. The choice of a two-chamber MFC with a liquid chemical catholyte, instead of the open-air cathode architecture is to focus the attention mainly into the new SSA. Considering the difficulty to estimate heat of combustion of wastewater, in literature the results usually show the Coulombic Efficiency (CE) that represents the conversion of organics into electrical charge.

However, CE does not take into account the energy expenditure to run the device and hence, it is not able to give indication of how far MFCs are from an energy-positive device^29^. Data present in literature, show CE values ranging from 1% to up 80%^28^,^29^,^30^,^31^,^32^,^33^. These variations mainly depend on the organic substrate but also on design, operative conditions and microbiology.

It is evident, therefore, that, in many cases, if energy consumption of the external devices were taken into account, these efficiency values would be dramatically lower.

The present work fixes a new important step toward the development of a solid phase anolyte and its capability to function as *nutrient storage* available for bacteria and convertible into useful energy in the time, without any external nutrients addition. It acts as true chemical energy storing system able to release slowly its energy over time and significantly contributes to the autonomy of the resulting system. The easy preparation of SSA and the easy uptake of it from bacteria reveal that solid SSA in an original and convenient fuel for MFC. This is an important step toward the reduction of energy request, bringing a better net energy balance necessary to overcome the biggest bottleneck to marketability of MFCs. The convenience of maintaining a growing community of bacteria thanks to a high-density energy substrate could mean a potentially long-term source of energy with numerous applications: from remote energy source to the waste management practices as treatment of both water and solid waste. Taking into account that many electronic devices or sensors, i.e. temperature sensors, require 1.5 V as operative voltage or even less, i.e. piezoresistive sensor^26^ require ~1 V, one possibilities is to design a system at least of two MFCs connected in series and eventually with a capacitive storage system. The remote application could take advantage from natural environments, i.e. seawater. There bacteria can found a richer substrate releasing more electrons, permitting to power sensors in remote area. Moreover, the high efficiency of the device (63%) together with the zero contribution for anodic feeding is a crucial step toward a new clear route to portable and autonomous MFC devices for remote applications, cutting down, significantly, operative costs, energy requirements for the system maintenance and reducing hydraulic problems.

It is worthwhile to underline that the anodic chamber, after it was sealed, did not need any more feeding or hydration during the experiment neither reinoculation, giving rise to a really autonomous device from the anodic side.

Next important step could be the integration of air-cathode to replace the chemical catholyte, in order to push the research toward a complete autonomous system. The non-astonishing performance have not to restrain the opportunity of a further development, because SSA shows the way for a new generation of MFCs, that can, for example, be, realistically, employed in devices for remote areas or harsh environments monitoring, powering small devices, such as sensors.

## Methods

### MFC configuration

The MFC devices consist of two squared chambers: the anode and the cathode. Both compartments were made in Poly(methyl methacrylate) (PMMA) with dimensions 8 cm × 8 cm × 2 cm and separated by a cation exchange membrane (CEM, CMI 7000, Membranes International Inc.), as shown in Fig. 1a. A square piece of graphite felt (64 cm^2^) (Soft felt SIGRATHERM GFA5, SGL Carbon, Germany) was introduced in both anode and cathode chambers and connected with a graphite rod (5 mm in diameter) to ensure an effective current recovery. The solid phase anolyte contains in: 8 g·L^−1^ sodium acetate, 7 g·L^−1^ glucose, 7 g·L^−1^ fructose, 10 g·L^−1^ peptone, 25 g·L^−1^ agar, 8.2 g·L^−1^ Na_2_HPO_4_·2H_2_O and 5.2 g·L^−1^ NaH_2_PO_4_·H_2_O dissolved into diluted seawater (ratio seawater: distilled water = 1:4). The solution, after being autoclaved at 125 °C for 15 min was poured into plastic mould, becoming solid overnight. The conductivity of SSA of 31.0 mS/cm was measured by WTW Multi 3430 multimeter (Germany).The electrolyte is, afterwards, released from the mould and placed into the anodic chamber. The gelling agent used, agar (Fig. 1b) is a polysaccharide, obtained from particular types of seawood and among the advantages that if offers for use in microbiology, the principle one is that it remains solid at normal incubator temperature, from 4 to 45 °C. In Fig. 1c is shown the picture of obtained SSA. Then, the SSA was inserted inside the anode chamber covering whole its volume saved for the carbon felt that was laid down upon it and the graphite rod and immersed into buffer solution. MFC was inoculated in the anode chamber by sea water (Arma di Taggia (IM), Italy), previously enriched with following cultures (in three steps) in anaerobic conditions. The experiment was performed at room temperature (24 ± 2 °C). The mixed culture, before being inoculated in MFCs was previously enriched in batch and under gentle orbital shaking (150 rpm), with the same medium used for SSA except for the absence of agar. This medium used for bacteria growth and inside the MFC is less selective in order to allow growing of resident marine consortia. Seven ml of enriched sea-water inoculum was directly distributed on carbon felt surface. To prepare anodic and cathodic solutions, a phosphate buffer, i.e. Na_2_HPO_4_·2H_2_O (8.2 g·L^−1^) and NaH_2_PO_4_·H_2_O (5.2 g·L^−1^) was used. The cathodic compartment was filled by potassium ferricyanide (6.58 g·L^−1^) used to perform reduction reaction. The electrolyte was replaced two times a week by syringe pump (NE-300 Just Infusion™ Syringe Pump, USA) during the experiments to ensure a constant behavior of the cathodic side of the cell. Regarding the anode, no need of replenishment or reinocultaion during all period of test was necessary, at all.

### MFC operation and characterization

From the electrical point of view, the experiment was divided into two phases (Fig. 2): during the first one (lasted from day 0 to day 15), the MFCs were investigated under a constant value of electrical loading (1000 Ohm). In the second phase (from day 15 to day 30) fifteen loads, from 330 to 8800 Ohm, from the highest to the lowest were sequentially applied (one per day. The cell voltage (ΔV) under external resistors was continuously measured every 60 s using a data logger (Agilent, 34972A, The Netherlands). During both phases, two-times a week, the external applied resistance was removed in order to perform an electrochemical characterization analysis, by means of a multi-channel VSP potentiostat (Biologic), by varying 1 mV/s potential from open circuit voltage to short circuit current conditions (linear sweep voltammetry technique-LSV), after a stabilization period (few minutes) under OCV conditions. Measurements were recorded by using EC-Lab® software version 10.1x (BioLogic) for data acquisition. Before starting the electrochemical measurements, the cathodic electrolyte was replaced by a fresh one. The cell performance was investigated calculating the power produced by the device for each load, by applying the Joule’s Law, P = V^2^/R, where R is the external resistance and V is the correspondent collected voltage between the anode and the cathode. The current interrupt method (CIM) was performed to determine the internal resistance of the MFC through the interruption of the current flow at fixed potential of 0.3 V and the observation of the resulting voltage transients. The test was carried out using a perturbation to the system with a very short duration (50 ms) in order to obtain precise and accurate determinations.

A calculation of the energy before (E_initial_) and after one-month operation (E_final_) was made considering the heat of combustion of the organic substrates embedded into SSA (in particular the lower heating value was taken into account). This measure was performed by isoperibolic calorimeter (Parr 6400, FKV Italy). By the difference between E_initial_ and E_final_ the energy consumed by bacteria’s metabolism in one month (E_consumed_) was obtained. In order to estimate the energy produce by MFC during the experiment, ΔV acquired by data logger was use to calculate the current (*I*) from Ohms law: *I* = *V*·*R*^−1^. Thus, energy produced (E_MFC−OUTPUT_) by SSA-MFC in Joule (J) during one-running month was calculated as the integration of the electrical power in the time (t) by the following equation:


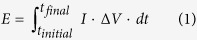


From these data, it was possible to estimate the energy efficiency (η) as the ratio between the E _MFC−OUTPUT_ and the energy consumed by microorganisms from SSA in one month:


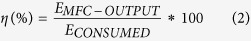


## Additional Information

**How to cite this article**: Tommasi, T. *et al*. New insights in Microbial Fuel Cells: novel solid phase anolyte. *Sci. Rep*. **6**, 29091; doi: 10.1038/srep29091 (2016).
